# Identification of Exo-miRNAs: A Summary of the Efforts in Translational Studies Involving Triple-Negative Breast Cancer

**DOI:** 10.3390/cells12091339

**Published:** 2023-05-07

**Authors:** Jarline Encarnación-Medina, Lenin Godoy, Jaime Matta, Carmen Ortiz-Sánchez

**Affiliations:** Department of Basic Sciences, Ponce Research Institute, Ponce Health Sciences University, Ponce 00716-2347, Puerto Rico; jencarnacion@psm.edu (J.E.-M.); lgodoy@psm.edu (L.G.); jmatta@psm.edu (J.M.)

**Keywords:** breast cancer, exosomes, exosomal microRNAs, triple-negative breast cancer, prospective studies

## Abstract

Triple-negative breast cancer (TNBC) accounts for about 10–15% of all breast cancers (BC) in the US and its diagnosis is associated with poor survival outcomes. A better understanding of the disease etiology is crucial to identify target treatment options to improve patient outcomes. The role of exo-miRNAs in TNBC has been studied for more than two decades. Although some studies have identified exo-miR candidates in TNBC using clinical samples, consensus regarding exo-miR candidates has not been achieved. The purpose of this review is to gather information regarding exo-miR candidates reported in TNBC translational studies along with the techniques used to isolate and validate the potential targets. The techniques suggested in this review are based on the use of commercially available materials for research and clinical laboratories. We expect that the information included in this review can add additional value to the recent efforts in the development of a liquid biopsy to identify TNBC cases and further improve their survival outcomes.

## 1. Introduction

**Figure 1:**
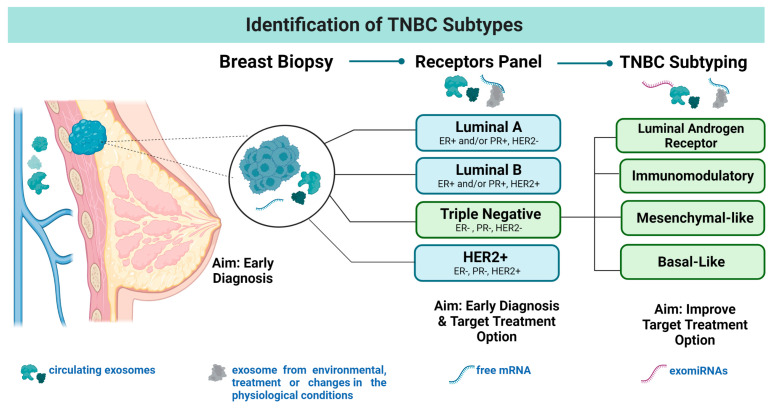
Identification of TNBC Subtypes. Breast cancer is a complex disease divided into four principal molecular subtypes. In addition, the TNBC subtype can be stratified into four additional subtypes including: (i) luminal androgen receptor (LAR), (ii) with immunomodulatory (IM) protein expression, (iii) having mesenchymal features (MES, mesenchymal-like), or (iv) BLIS (basal-like and immune suppressed). Segments highlight the presence of the exosomes parallel to the screening process.

Triple-negative breast cancer (TNBC) accounts for about 10–15% of all breast cancer (BC) cases in the US alone and its diagnosis has been associated with poor survival outcomes [1]. A better understanding of the disease etiology is crucial to identify target treatment options and improve patient outcomes [2]. During the past two decades, scientists have studied and reported the pathological differences within triple-negative (TN) tumors [3]. Currently, TNBC is stratified into four major subtypes based on their pathological and genetic characteristics [2,3,4,5,6]. However, additional classifications are available based on gene expression and other molecular features of TN tumors [7]. Bou Zerdan et al. (2022) summarized the evolution of the TNBC classification gathering features from basic subtyping, genetic expression, and treatment options/response (Figure 1). TNBC stratifications include: (i) tumors with androgen receptors (LAR, luminal androgen receptor), (ii) tumors with immunomodulatory (IM) protein expression, (iii) tumors with mesenchymal features (MES, mesenchymal-like), or (iv) BLIS (basal-like and immune suppressed) [8]. The BLIS subtypes are characterized for their enrichment in proteins related to the cell cycle [7].

From a clinical perspective, several factors have been suggested to be potential contributors to these poor survival outcomes including the lack of appropriate clinical treatment and delays in diagnosis [4,5]. The importance of early detection was demonstrated using data from the US Surveillance, Epidemiology, and End Results (SEER) program and multi-cancer early detection (MCED) tests [9]. Hubbell and co-workers, using a mathematical approach, were able to prove that early detection can reduce cancer-related mortality [9]. The public sectors also agree that early diagnosis can be an important factor to prevent cancer-related deaths [10,11]. Early detection provides additional time for the physician to explore options within recent treatments for the disease, in this case, TNBC. Thereby, the ongoing effort to develop a liquid biopsy using exo-miRs expression as a stand-alone or as a companion test to improve TNBC detection has great potential for improving disease outcomes [12].

This article aims to summarize the translational research studies using blood-derived exo-miRs to define TNBC features. In addition, summarized information regarding basic exo-miR biology and the commonly used extraction techniques for liquid biopsy applications in the BC field is also included. Important topics regarding exosomal extraction are also discussed including: (i) markers for exosome identification and (ii) potential sample contaminators. Within the benefits of using exo-miRs to develop a liquid biopsy are: (1) their stability at 4 °C in a neutral pH which is cost-effective, (2) their high abundance in the blood compartments (serum or plasma), and (3) the wide variety of extraction methods available [13,14]. Further research is needed to take advantage of the exo-miRs abundance in blood (Figure 1). In terms of cancer, a deeper understanding of the applicability of exo-miR levels is crucial to improve their ability to detect the malignancy and avoid confusion due to the general miRNA production resulting from normal metabolism. Our goal is to provide a description of the existing exo-miR translational studies conducted in the field BC and to highlight the studies performed in TNBC cohorts.

## 2. Materials and Methods

The literature search was performed using the PubMed database (https://pubmed.ncbi.nlm.nih.gov/about/) (accessed 20 March 2023). Initially, 46 articles were identified using the search terms ((exosomal) AND (microRNA)) AND (triple-negative breast cancer). However, only articles aiming to develop an exo-miR-based liquid biopsy tool for TNBC diagnosis through cohort studies were included within a publication date range of ten years (from 2013 to 2023). Only studies using patients’ plasma or serum were considered. In addition, studies using exo-miRs to elucidate TN tumor features were also included.

## 3. Exo-miR Biology: Formation and Role in TNBC

miRNAs are small non-coding RNAs that regulate gene expression at a post-transcriptional level. The biogenesis of these molecules is widely documented [15,16,17]. In general, these molecules’ journey starts at the cell nucleus where they are transcribed as primary miRNA (pri-miRNAs) by RNA polymerase II (Poll II). The resulting hairpin-shaped molecule is transformed into a pre-miRNA molecule with 60–70 nucleotides after binding with DROSHA/DGCR8 complex. The pre-miRNA molecule leaves the nucleus through an exporting protein (XPO5) to later become a mature miRNA by chemically interacting with a ribonuclease III enzyme (DICER1). This mature miRNA becomes part of the miRNA-induced silencing complex (miRISC) to regulate post-transcriptional modifications or to be secreted from the cell lumen. The cells release exosomes along with miRNAs by endosome-exosome mechanisms to activate apoptotic pathways to prevent cancer while maintaining homeostasis [18,19,20,21]. Exosomes regulate key physiological functions in healthy individuals such as angiogenesis [22,23] and immune function [24,25], among others. However, exosomes also have different roles in diseases such as cancer [13,26]. As an example, in colorectal cancer (CRC), the hypoxic tumor microenvironment has been found to lead to exosome secretion. Ge et al. (2021) found that hypoxic CRC cells can promote G1-S cycle transition and proliferation while preventing the apoptosis of tumor cells. This is achieved by transmission of miR-210-3p through exosomes from hypoxic to normoxic tumor cells [27]. These facts highlight the crucial role of exosomes in cellular communication in healthy and non-healthy individuals.

**Figure 2:**
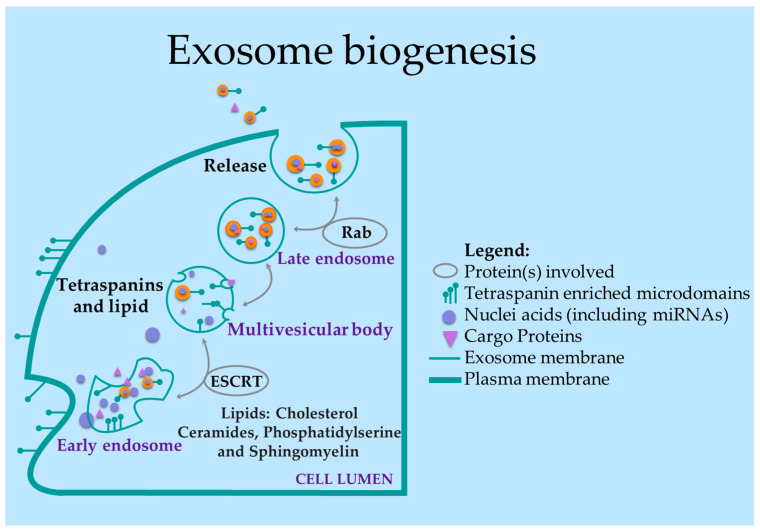
Exosome biogenesis showing the transport of mature miRNAs to the extracellular compartment. The early endosome is a result of the inward budding of the cell membrane into the cellular lumen. The protein complex ESCRT and other molecules contribute to the development of the multivesicular body that is later transported by Rab protein to be released from the cellular lumen.

According to the International Society of Extra-cellular Vesicles, their size can range between 50 to 100 nm [28]. Other molecules included in the exosomal cargo are: fragmented or intact mRNA, long non-coding RNA, ribosomal RNA (rRNA), or small non-coding RNA (18–23 nt) [29,30]. Extracellular vesicle secretion, and thereby exosome secretion, mainly relies on the coordination of the endosomal sorting complex required for transport (ESCRT) [31]. Other proteins involved in exosome secretion are tetraspanins (i.e., CD63, CD9, CD81, CD82) and MHC-I and MHC-II [32]. A scheme for exosome biogenesis is present in Figure 2.

In terms of BC, studies have reported the presence of the required protein complexes to produce the exo-miRs in BC cells. However, significant variations in DICER or DROSHA gene expression have been reported, where these proteins are mostly down-regulated in TNBC [33,34]. This opens a new avenue to study the miRNA regulations in TNBC. Since exo-miRs have key roles in post-transcriptional modifications, their functions as cellular communication centers involved in genetic exchange between cells, and their preservation in body fluids make them a good target for a liquid biopsy [16]. Exo-miRs have also been found to regulate different aspects in BC such as: proliferation [35], chemoresistance [36], and tumor microenvironment [29,37].

### 3.1. Exosome Isolation and Evaluation of Exosomal Markers

Within the wide variety of extraction methods available, ultracentrifugation is the most acceptable in the exosome field despite its cost [13,14]. However, independently of the preferred extraction method further confirmation of successful exosome isolation can also be performed. This can be achieved by evaluation of the expression of exosomal proteins. Among the most frequently used “exosomal marker proteins” are components of the ESCRT and other accessory proteins [38]. This group of proteins includes: Alix, TSG101, HSC70, and HSP90β, which are expected to be found in exosomes independently of their cell of origin [31,39,40,41]. However, since some of these proteins could also be found in the cytosol [42,43,44], it would be difficult to distinguish their endogenous expression on the exosomes from cellular contamination during the exosome isolation process. Testraspanins (i.e., CD9, CD63, CD81) are also commonly used as specific exosome markers [45,46]. However, since tetraspanins are key components of the cell surface and the plasma membrane, they can also be detected in microvesicles. Microvesicles, which range from 50 to 1000 nm in diameter, are formed by outward budding from the plasma membrane [32]. Exosomes are considered to be highly enriched in tetraspanins with a 7- to 124-fold when compared to their content in the parental cells. Tetraspanins CD9, CD63, CD37, CD81, or CD82 are specially enriched in the membrane of exosomes, and they are often used as exosome biomarkers [32].

In order to rule out microvesicle contamination, some studies measure Arf6 protein expression on the exosome isolation product. Arf6 is a marker for microvesicles [47,48]. This approach was used by Patel et al. (2019) when comparing the efficacy of different exosome isolation methods [49]. In addition, some studies also include protein markers for cell organelles such as Golgi (i.e., GM130) [50,51] on their experimental design to assess the quality of the exosome isolation processes. These can serve as negative control markers or as markers of cellular contamination. In this case, including whole cell lysate for analysis of markers could be useful. By using specific exosome markers along with markers for cell contamination, a more precise assessment of the purity of the exosome fraction can be achieved. In addition to the commonly used exosome markers, exosomes can also express proteins that provide information regarding their cells of origin. Efforts have been made to establish specific breast cancer-associated exosome markers as presented in Table 1.

### 3.2. Special Considerations: Non-Exosomal Contamination and Data Normalization

To this day, there are a limited number of comprehensive clinical studies on exosomes and BC. One possible factor for this could be the limitations of having a proper standardized technique for the isolation of high-purity homogeneous extracellular vesicles (EVs) and their specific subtypes from physiological fluids. Differential ultracentrifugation is the more commonly used technique for exosome isolation, which separates and concentrates exosomes according to their density [59,60]. However, this technique has several drawbacks, including: the co-isolation of non-exosomal impurities, low reproducibility, low RNA yield, and potential damage of exosomes [61]. Functional studies performed with plasma-derived exosomes showed that functions of the isolated exosomes may be negatively influenced by “contaminating”, non-exosomal materials. These impurities include protein aggregates that can act as carriers of circulating miRNAs and protect them from RNase activity. Arroyo et al. (2011) demonstrated that Argonaute2 complexes can carry circulating microRNAs independent of vesicles in human plasma [62]. To overcome this problem, new techniques such as precipitation of EVs using polymers, and density gradient isolation techniques have emerged. However, additional purification steps might be needed to separate EV subpopulations from each other, from other micro-particles with similar densities, and from the density gradient matrix [59]. Kurian et al. 2021 summarize other isolation techniques based on the exosomes’ physical and chemical properties; however, due to the complexity of translational studies these types of techniques are not commonly used in the TNBC field [63].

Additional factors that impact the amount, purity and, heterogeneity of EVs from blood include: sample collection, handling, storage conditions, stability, anticoagulants, volume of blood collection, time of blood collection, and the age, sex, disease state, and fed/fast status of the animal/patient [64].

To further enhance the potential of exo-miRs as potential diagnostic and prognostic biomarkers, proper normalization techniques to assess differences in miRNA expression among study groups should be established. The utilization of internal controls such as small nuclear RNAs SNORD44 (RNU44), SNORD48 (RNU48), and the nuclear RNA RNU6-1 (U6) as reference elements for miRNA quantification in cell and tissue samples is extensively common [37,65,66]. However, there is no consensus on standard reference miRNAs for qRT-PCR normalization, especially for plasma exosomes [67]. The introduction of an exogenous miRNA as spike-in control has been proposed as a possible normalization strategy. Nevertheless, this approach only allows the control of technical biases related to sample preparation without ensuring the adjustment for biological and other technical variability factors. The use of algorithms, such as geNorm, NormFinder, and BestKeeper, have been developed to identify the most stable endogenous genes to use as reference, under a specific experimental condition [68].

## 4. Benefits of Including a Discovery Cohort with a Control Group in Translational Studies

The role of exo-miRs has been widely investigated in healthy individuals [69] and some exo-miRs have been reported to be a result of normal metabolism. Exo-miRs have been detected in breast milk, urine, and saliva among other fluid compartments in healthy subjects [12,30,70,71]. Researchers have also been able to find exosomes in the tears from healthy individuals [72]. Moreover, differences in exosome secretion due to gender and ethnic differences have been reported [73,74]. Therefore, including samples from controls (subject without the disease) within the discovery experiment is important since it will allow for detection of variations of miRNAs due to physiological changes.

### 4.1. Including a Control Group

Although case–control studies provide pivotal evidence to suggest that the excess of exosomes in blood samples is a product of cancer metabolism, the inclusion of individuals without cancer (control group) is highly recommended [75,76,77]. The inclusion and exclusion criteria for selection of the control group must be rigorous and implemented consistently among study participants. It is more frequent to find a control group on observational studies, nested studies, or studies involving consortiums. On the contrary, in prospective studies, where the objective is to investigate biomarker changes along with disease progression, the inclusion of controls is not always feasible. Although this type of experimental design is convenient to maximize laboratory resources and decrease the number of study subjects, including a control group can provide a clearer idea as to whether the observed changes are only related to disease progression.

### 4.2. Discovery Experiment

A discovery cohort is composed of a subset of samples representing the study groups (i.e., case–control groups). The sample selection for this cohort is usually performed from a pool of participants that match the study eligibility criteria tied to the potential clinical biomarker (i.e., BC subtype, tumor grade, among others). This cohort is used to perform a discovery experiment. In general, these discovery experiments are conducted using several array platforms. Pepe et al. (2011) provides insightful information regarding discovery experiment planning [78]. High throughput techniques to increase the number of experimental candidates are frequently used for this type of experiment [79,80]. Therefore, the batch to batch effect correction and the discovery sample size need to be evaluated [81,82]. Although the discovery experiment is expected to have a smaller sample size than the validation experiment, it is important to include a reasonable amount of samples. Table 2 shows the most commonly used techniques for discovery experiments.

## 5. Current Efforts on the Development of a Liquid Biopsy Tool for TNBC Detection

Within the most recent publications involving BC and exo-miRs there are at least seven key studies, to our knowledge, which are focused on TNBC detection/diagnosis (Table 3). In general, most of the studies began with a discovery experiment using a high-throughput technology followed by a validation cohort. This implies increasing the sample size of the study groups and often using another technique to measure the expression of the target(s). In terms of biomarkers, it is very important that the validation cohort accounts for proper sample size, instead of using other technology [83]. From the studies related to BC and TNBC, we observed that most of the studies consistently followed this method (Table 3). In vitro models were also included in some experimental designs whereas others included data from previously published observational studies to justify their interest to investigate a specific exo-miR candidate.

The use of published data is another strategy to choose candidates or narrow down the number of targets from the discovery experiment and can be supported by an in silico analysis. A wide number of articles are available to support the use of these algorithms in these online platforms [84,85,86,87,88,89]. Comparisons among the different platforms and insight into how to interpret their results are also available for public review [89,90,91].

In terms of exo-miR extractions, most of the investigators used precipitating solutions and only one study reported the exosome extraction using the ultracentrifugation method [92]. The real-time polymerase chain reaction (RT-PCR) technique was used to detect the exo-miR targets on the validation cohorts in almost all the studies regardless of the differences in the extraction methods (Table 3) [93].

The most recently published study involving TNBC and exo-miRs was published in 2020 [76]. The discovery experiment from this study team was based on the use of next-generation sequencing to select the exo-miRs related to BC subtypes and then confirm the results through RT-PCR. A prospective experimental design was then employed, through a 2-year follow-up to determine whether the patients had recurrence. The initial study cohort was composed of 30 treatment-naïve participants (controls = 3, BC cases = 27). Variations on the expression of 54 exo-miRs were detected in TNBC samples (*n* = 6) when compared to the control group (*n* = 3). Validations through RT-PCR were performed focusing on eight targets using a small cohort of 40 subjects (cases = 20, controls = 20). These targets were chosen based on a bioinformatics analysis using Gene Ontology (GO) and Kyoto Encyclopedia of Genes and Genomes (KEGG) databases [94]. When samples from controls and women with TNBC were compared: miR-148a-5p, miR-200a-5p, miR-210a-3p, miR-378a-3p, miR-483-5p, and miR-7110-5p were upregulated while miR-92b-3p and miR-150-5p were downregulated. The results obtained from the second experimental set show an upregulation in exo-miR levels in BC patients with recurrence when compared with patients without recurrence: miR-150-5p (AUC = 0.705), miR-576-3p (AUC = 0.691), and miR-4665-5p (AUC = 0.681). Exo-miR-150-5p was observed on the initial analysis; however, no significant differences were found in the RT-PCR results. This might be explained by its capacity to increase along with the disease progression since exo-miR-150-5p was present in patients that had recurrent TNBC. Variations on the regulatory role of the miR-150-5p have been reported on other BC studies, although more studies involving the exo-miR are needed [95,96].

In 2018, four articles involving exo-miRs and TNBC were published. An elegant experimental design was published by Stevic and co-workers [75] using samples obtained from the GeparSixto trial [97,98]. In this observational study, the team was able to collect blood samples from BC patients (*n* = 435: TNBC, *n* = 224, and HER2+, *n* = 221). The discovery experiment (*n* = 15) was focused on TNBC considering the treatment status (carboplatin) along with the pathological complete response (pCR). The exo-miRs from the discovery experiment and the validation cohort were identified using TaqMan microRNA array cards. The first array, which consisted of 348 targets was performed in a small cohort of TNBC patients (*n* = 15). A total of 45 candidates were chosen to conduct a larger study including 435 patients including samples representative of the HER2 subtype. miR-199a, miR-125, miR-193b, miR-365, and miR-370 were included in the larger cohort due to the variability in their expression levels. The miRs that were found to be significantly different when comparing cases and controls and controls with the subtypes groups were: miR-30c, miR-150, miR-152, miR-199a, miR-340, miR-410, and miR-598. Among these candidates, miR-199a-3p was associated with tumor size in all cases while miR-410 was exclusive for the HER2 subtype. miR-30c was associated with tumor grade within the TNBC subtype. Since the focus of this article is exo-miRs as a liquid biopsy, therapeutic outcomes will be not discussed in detail.

A different list of exo-miR candidates was presented by the study of Ni et al. (2018), including: miR-16, miR-30b, and miR-93 [99]. As well as the previously described studies [75], this group used the TaqMan miRNA array card assay for target identification followed by RT-PCR validation. The results from both experiments were partially overlapping. The trend of the results was similar, but the significance levels were not the same. The previous fact underscores the importance of having a validation cohort with a robust sample size. Patients with BC, ductal carcinoma in situ (DCIS), and controls were evaluated on both experimental sets. High levels of miR-16 were reported on samples from BC cases when compared to the controls. miR-16 has been used as a reference miR due to its stability after freezing and thawing processes [100]. Previous studies have demonstrated a null variation between samples from cases with different cancer types and controls [101]. However, the use of specific reference miRs, such as miR-16, does not automatically apply to all studies, as previously mentioned. Ni et al. (2018), also reported that miR-16 levels were high in patients with estrogen receptor (ER) positivity when compared to the TNBC group. Increasing levels of miR-93 were detected on the DCIS group when compared to the other groups suggesting that this candidate might be appropriate to track the disease progression. The miR-93 has been also used as a reference miR in the past [101]. The exo-miR-30b was also found in higher levels in the DCIS group when compared to the controls and all BC cases. Surprisingly, the exo-miR-30b has lower expression in patients that experience recurrence when compared to DCIS. Additionally, significant results were obtained comparing lobular and tubular tumor types.

Li et al. (2018) used an experimental design based on identifying the miRs related to BC following the same criteria proposed in this review by including a discovery and a validation cohort. The detection of the exo-miRs was considered an external validation. miR-20b-5p and miR-106a-5p were detected on plasma and serum and were included in the external validation. miR-106a-5p was upregulated in exosomes from both compartments: serum and plasma. The expression levels of miR-106a-5p and miR-20b-5p were reported in patients with low histological grades, ER-positive, and HER2-negative status according to an association test. It is worth mentioning, as also highlighted by the co-authors, that miR-106a-5p can be a potential candidate for BC early diagnosis. Although TNBC samples were included in the study, no results were presented by the team [102].

Another translational study worth attention in the TNBC field was published by Eichelser et al. (2014). The targets were partially chosen based on previously published data including a manuscript published by the study team [103,104,105]. The targets studied were: miR-101, miR371, miR-372, and miR-373. Additionally, in vitro studies were performed to shed light on the cellular mechanism that might explain one of the molecular roles of miR-373 in BC cells. The quantification of serum cell-free miRs (miR-101, miR-372, and miR-373) was performed in study participants with invasive BC, benign breast disease, and controls. Significant dysregulation was detected on the expression of miR-101 and miR-373. The same candidates were evaluated using serum from 50 BC patients and 12 controls. Comparisons were established between the exo-miRs and the cell-free miRs results. Regarding the TNBC group, the enriched exosomal serum fraction had higher levels of miR-373 when compared to the cell-free miRs. This was consistent when comparisons among Luminal, HER2+, and controls were performed [106].

A combination experimental design using in vitro assays, animal models, and patient samples was employed by Hannafon et al. (2016). Cell lines representing different BC subtypes were used including: luminal A (MCF7), TN (MDA-MB-231), and non-tumorigenic (MCF-10A) cell lines. The initial experiment was focused on studying the exosome content from the supernatant vs. the cellular content. miR-122 and miR-451 were differentially expressed in the luminal A subtype model while miR-1246 was detected on both cell lines: MCF-7 and MDA-MB-231. The animal experiments were conducted using patient-derived xenografts (PDX). The expression miR-1246 was higher in mice representing any of the BC subtypes, although mice with TN tumors showed a higher abundance of this miR. miR-451 was undetectable in PDX mouse plasma. A small cohort including 36 patients was used to further confirm these results. High levels of miR-1246 were reported in plasma exosomes from BC patients while miR-122 was no longer significantly expressed. Exo-miR-21 was also reported as highly expressed on the plasma samples from BC patients [77].

Lastly, the potential of the exo-miR-223-3p to discriminate between BC type (invasive and in situ components) was also investigated by Yoshikawa et al. (2018). The discovery experiment was based on the study of 2565 exo-miR targets in BC patients with in situ and ductal carcinoma and controls. From these experiments, 5 exo-miRs were significantly different among the study groups, including: miR-223-3p, miR-130a-3p, miR-191-5p, miR-146a, and miR-221-3p. The main target was chosen based on having a significantly higher exo-miR fold-change among groups. This candidate was tested using in vitro assays by transfecting miR-223-3p and evaluating the effects of its expression over proliferation and invasion in MCF-7 cells. Although samples from the different subtypes were considered, the study was focused on the luminal A subtype. Yoshikawa et al. (2018) found a positive correlation between exo-miR-223-3p expression in blood and tissue samples from the same patients. This correlation and the results from the in vitro experiments suggest that exo-miR-223-3p can be associated with the presence of the tumor. Lastly, the study team found an association between having an invasive ductal carcinoma (IDC) and upstage IDC (stage 1) and high levels of miR-223-3p when comparing with a non-advanced DCIS disease. Their results suggest that miR-223-3p is a potential candidate to further study by increasing the number of individuals [107].

**Table 3:** Selected studies focused on the identification of exo-miRs in BC and the TN subtype.

Study	Discovery Experiment	Validation Cohort	Isolation Method	Detection Method	Study Results
Wu et al. (2020) [76]	Yes (*n* = 30)	Yes (*n* = 40)	Exosome Isolation Reagent	Next generation sequencing and RT-qPCR	Upregulated miRNAs were miR-148a-5p, miR-200a-5p, miR-210a-3p, miR-378a-3p, miR-483-5p and miR-7110-5p). Downregulated miRNAs were: miR-92b-3p and miR-150-5p.
Stevic et al. (2018) [75]	Yes (*n* =15)	Yes (*n* = 455)	ExoQuick Exosome Precipitation Solution	TaqMan microRNA array Human Pool A cards and TaqMan RT-PCR	Significant differences on 31 of the targets were detected among BC subtypes (HER2+ and TNBC). A significant association was found between exo-miR expression levels and tumor characteristics.
Ni et al. (2018) [99]	Yes (*n* = 48)	Yes (*n* = 192)	ExoQuick Exosome Precipitation Solution	TaqMan miRNA array cards and TaqMan miRNA assays	miR-16, miR-30b, and miR-93 have specific exosome packaging. Levels of miR-93 were significantly enriched in exosomes from DCIS patients rather than BC patients. Levels of miR-16 were high in patients with ER+ (*n* = 85) when compared to TNBC patients (*n* = 24).
Li et al. (2018) [102]	Yes Plasma (*n* = 400) Serum (*n* = 406) samples	Yes (*n* = 32) Exosome extraction	ExoQuick Exosome Precipitation Solution	qRT-PCR	miR-20b-5p was significantly upregulated in BC. Exo-miR-106a-5p was consistently reported across compartments. No potential results related to TNBC subtype were reported, although BC subtypes were considered in the experimental design.
Eichelser et al. (2014) [106]	Yes (*n* = 215)	Yes (*n* = 62)	ExoQuick Exosome Precipitation Solution	TaqMan MicroRNA Assays	miR-101, miR-372, and miR-373 were found in higher expression in the exo-miR fraction when compared with cell-free miRNAs. miR-373 was highly expressed on TNBC samples
Hannafon et al. (2016) [77]	Combination of in vitro and PDX mice	Yes (*n* = 32)	ExoQuick Exosome Precipitation Solution	qRT-PCR	miR-1246 and miR-21 were significantly highly expressed on BC patients. ROC: miR-1246 (0.69), miR-21 (0.69), and the combination (0.73).
Yoshikawa et al. (2018) [107]	Yes (*n* = 9)	Yes (*n* = 199)	Ultracentrifugation	TaqMan RT-qPCR	exo-miR-223-3p was higher in BC cases and controls. Exo-miR-223-3p expression was associated with the histological type, pT stage, pN stage, pathological stage, lymphatic invasion, and nuclear grade.

## 6. The Use of In Vitro Models to Study Exo-miR Mediated Mechanisms

In order to study the role and function of exosomes in cancer, different types of in vitro studies can be performed [36,108]. The most common experimental design setup consists of isolating the exosomes from the cells of interest, followed by characterization and quantification, to be cocultured with the potential target cells in order to assess the effect of the exosomes over key biological endpoints. As an example, Gernapudi et al. (2015) studied the effect of exo-miRs secreted from mouse preadipocytes over MCF10DCIS cells (an early stage BC model). This group aimed at elucidating how preadipocyte-derived exosomes can regulate early stage BC through stem cell renewal, cell migration, and tumor formation. Their results show that the exo-miR-140/SOX2/SOX9 axis can regulate differentiation, stemness, and migration in the tumor microenvironment. This highlights the crucial role of exosomal signaling over the tumor microenvironment [109].

A study by Santos et al. (2018) found that miR-155 can be transferred through exosomes to confer chemoresistance to recipient cells. This group found increased levels of miR-155 in exosomes secreted by cancer stem cells and Doxorubicin (DOX) and Paclitaxel (PTX) resistant cells [36]. When these exosomes were cocultured with sensitive cells, a strong induction of miR-155 levels was observed in recipient cells suggesting that this exo-miR can be transferred from cell-to-cell through exosomes. In addition, the transfer of miR-155 increased the migration potential of recipient cells and conferred cells increased resistance to DOX and PTX. These two studies provide an idea of the relevance of the performance of in vitro studies in order to further understand the biological roles of exo-miRs in BC.

Although coculture methods are used to resemble direct or indirect cellular interactions, they have the limitation of lacking the complexity of an individual’s metabolism. In terms of cancer translational studies, it is difficult to avoid contamination from exosomes that are released from metabolic processes. In vitro studies allow the researchers to elucidate the biological explanation of the potential role of exo-miRs in specific cells. These can be implemented as part of population studies since they provide a mechanistic understanding of the biological parameter that is being studied in the population.

## 7. Future Perspectives to Advance TNBC and Liquid Biopsy

Prospective research studies are challenging to design and require a great amount of resources and the ability to account for multiple confounders from the beginning of the study [110]. This type of study implies the follow-up of a cohort and establishing a workflow for sample collection taking into consideration the study power [111]. In terms of BC research aiming to develop a liquid biopsy, more prospective studies are needed to understand the changes in exo-miR expression levels during disease progression and treatment.

Exo-miRs can provide researchers with the potential to track the changes once the malignancies are developed. During the experimental design phase, it is essential to include a control group (cancer-free individuals) and to consider the treatment status from the cases since this will also be reflected on the study results. As previously mentioned, experts in the extracellular vesicle field may require other experiments to further confirm the presence of exosomes (30–150 nm) before performing the exo-miR extraction [38]. It is also important to keep in mind that the US government and Clinical Laboratory Improvement Amendments (CLIA) might have specific requirements regarding accuracy and precision in order to validate a clinical test for public use [112].

In terms of the advances in the use of exo-miRs as a liquid biopsy tool for TNBC, the field has outstanding potential for further development. The previously presented studies highlight the importance of improving BC diagnosis by finding a biomarker able to: discriminate among BC subtypes (i.e., miR-1246), determine the probability of a DCIS to become an IDC (i.e., miR-223-3p), predict tumor grade (i.e., miR-20a), to study the pCR (i.e., miR-301), and to estimate the probability of recurrence (i.e., miR-150-5p miR-576-3p, and miR-4665-5p). These studies reflect an experimental design based on collaborative work to maximize resources. Most importantly, these studies are aiming to shed light on the exo-miRs expressed on BC overall but more specifically, on TNBC [3]. Early diagnosis in BC can positively impact the patients’ disease-free survival, especially in women with TN tumors, independently of their culture [113,114,115]. Ginsburg and co-workers explain in depth the benefits of the early detection of BC and the current efforts to implement it in different countries [116].

Another gap on the applicability of these liquid biopsy tools is regarding the ethnicity of the study participants. Evidently, additional efforts are needed to include minority ethnic groups (i.e., Hispanic/Latinos) in the study design to evaluate the exo-miR expression profile in BC and among BC subtypes. Since the exo-miR field is in constant development, it is a challenge to stipulate an ideal experimental design to successfully develop a liquid biopsy tool [117].

## 8. Conclusions

Currently, there is no standard procedure to follow as a reference of success in finding a tumor biomarker, especially in BC. This is primarily due to the complexity of this disease and its further stratification into different molecular subtypes. The issue of potential confounders such as treatment status must also be considered. Since TNBC can also be further stratified within various subtypes with different prognosis, additional challenges lie ahead for the understanding of this disease and the development of liquid biopsy tools. We expect that our effort of gathering the results and discussing the methods from published articles will allow others to construct their study design while the field moves to a harmonized workflow to study the exo-miRs in cancer patients establishing cutoff values.

## Figures and Tables

**Table 1:** Breast cancer specific exosomal markers detected in clinical samples.

Exosomal Markers	Body Fluid	Isolation/Detection Method	Ref.
CD24	Serum	Ultracentrifugation/Western blot	[52]
Survivin, Survivin-ΔEx3, Survivin-2B	Serum	ExoQuick/Western blot	[53]
HER2	Serum	Ultracentrifugation/Western blot	[54]
EpCAM, HER2	Plasma	Immunocapture on microfluidic chip/Immunofluorescence	[55]
Glypican-1	Serum	Ultracentrifugation/FACS	[56]
Periostin	Plasma	Ultracentrifugation/Western blot	[57]
CD47	Serum	Total Exosome Isolation kit/Flow cytometry and ELISA	[58]

**Table 2:** Advantages and disadvantages of high throughput exosomal miRNA detection methods.

Exo-miRNA Detection Kit	Pre-Amplification Step	Expertise in Bioinformatics Required	Normalization	Internal Controls	Cost- Effective
TaqMan MicroRNA array Human Pool A Card	Yes	No	The most suitable reference miR is selected from the experimental data.	One	Yes
nCounter^®^ miRNA Expression Panels	No	No	miRs for normalization are included by the manufacturer.	Included in the assay by the manufacturer	Yes
Next generation sequencing	Yes. Library construction	Yes	Reads are mapped with a genome reference sequence (miRBase).	Included in the assay	No
